# A Combined Supplementation of Omega-3 Fatty Acids and Micronutrients (Folic Acid, Vitamin B_12_) Reduces Oxidative Stress Markers in a Rat Model of Pregnancy Induced Hypertension

**DOI:** 10.1371/journal.pone.0111902

**Published:** 2014-11-18

**Authors:** Nisha G. Kemse, Anvita A. Kale, Sadhana R. Joshi

**Affiliations:** Interactive Research School for Health Affairs (IRSHA), Bharati Vidyapeeth Deemed University, Pune, India; University of Iowa, United States of America

## Abstract

**Objectives:**

Our earlier studies have highlighted that an altered one carbon metabolism (vitamin B_12_, folic acid, and docosahexaenoic acid) is associated with preeclampsia. Preeclampsia is also known to be associated with oxidative stress and inflammation. The current study examines whether maternal folic acid, vitamin B_12_ and omega-3 fatty acid supplementation given either individually or in combination can ameliorate the oxidative stress markers in a rat model of pregnancy induced hypertension (PIH).

**Materials and Methods:**

Pregnant Wistar rats were assigned to control and five treatment groups: PIH; PIH + vitamin B_12_; PIH + folic acid; PIH + Omega-3 fatty acids and PIH + combined micronutrient supplementation (vitamin B_12_ + folic acid + omega-3 fatty acids). L-Nitroarginine methylester (L-NAME; 50 mg/kg body weight/day) was used to induce hypertension during pregnancy. Blood Pressure (BP) was recorded during pregnancy and dams were dissected at d20 of gestation.

**Results:**

Animals from the PIH group demonstrated higher (p<0.01 for both) systolic and diastolic BP; lower (p<0.01) pup weight; higher dam plasma homocysteine (p<0.05) and dam and offspring malondialdehyde (MDA) (p<0.01), lower (p<0.05) placental and offspring liver DHA and higher (p<0.01) tumor necrosis factor–alpha (TNF–ά) levels as compared to control. Individual micronutrient supplementation did not offer much benefit. In contrast, combined supplementation lowered systolic BP, homocysteine, MDA and placental TNF-ά levels in dams and liver MDA and protein carbonyl in the offspring as compared to PIH group.

**Conclusion:**

Key constituents of one carbon cycle (folic acid, vitamin B_12_ and DHA) may play a role in reducing oxidative stress and inflammation in preeclampsia.

## Introduction

Preeclampsia (PE) is widely believed to be of placental origin [1], [2] and a common cause of maternal morbidity and mortality [3]. Inspite of considerable research the aetiology of PE remains elusive [4]. The role of maternal nutrition in influencing the risk of developing preeclampsia is unclear. Some studies suggest that supplementation with nutrients like calcium [5], [6] in the treatment of preeclampsia have beneficial effects. On the other hand other studies suggest that dietary supplementation with calcium [7] and dietary intake of magnesium [8] do not aid in reducing the risk of preeclampsia.

Maternal vitamins and minerals have been shown to influence angiogenic factors in PE [9]. Higher risk of preeclampsia in women with higher homocysteine and lower folate concentrations and vitamin B_12_ levels has been reported [10]–[13]. In contrast, other studies indicate no association of vitamin B_12_ with preeclampsia [14]–[16]. Further folic acid supplementation studies are inconsistent with some indicating beneficial effects [17]–[20] and others indicating no benefits [21]–[24].

Similarly epidemiological studies indicate a negative association of n3 fatty acids with risk for PE [8], [25]. Literature suggests that fish oil supplementation may be beneficial in reducing risk of preeclampsia [25], [26]. A recent review suggests that maternal dietary ω-3 PUFA supplementation limits placental inflammation and oxidative stress [27] although limited data is available on its effects on PE [28]. Recent reports suggest that inflammation and oxidative stress play a role in the pathophysiology of preeclampsia [2], [29]–[31]. Micronutrients like folic acid and vitamin B_12_ are important determinants of the one carbon cycle and play a critical role in determining pregnancy outcome [32]. Considerable experimental evidence indicates that micronutrient deficiencies or supplementation can modulate immune and inflammatory responses [33]–[35]. We and others have extensively demonstrated that these micronutrients and omega-3 fatty acids such as DHA are interlinked in the one carbon cycle and influence epigenetic changes in the placenta [36]. Omega-3 fatty acids, such as eicosapentaenoic acid (EPA) and docosahexaenoic acid (DHA), are known to have anti-inflammatory effects [37].

A series of our studies have shown that altered folate, vitamin B_12_ levels and reduced DHA levels leads to increased homocysteine and oxidative stress in preeclampsia [38], [39]. We hypothesize that combined supplementation of micronutrients (folate and vitamin B_12_) and omega-3 fatty acids may reduce the inflammatory cytokine like tumor necrotic factor – alpha (TNF-α) in a rat model of pregnancy induced hypertension.

The objective of the study was to examine the effect of various nutrient supplements i.e. folic acid, vitamin B_12_ or omega-3 fatty acids given individually or in combination during preeclampsia in reducing inflammatory cytokine using a rat model of pregnancy induced hypertension.

## Materials and Methods

The present study was carried out in accordance with the CPCSEA guidelines (Committee for the purpose of control and supervision of experimental animals) Govt of India. This study was approved by the Bharati Vidyapeeth Animal Ethical Committee (IAEC/CPCSEA/2618). The institute is recognized to undertake experiments on animals as per the CPCSEA, Govt of India.

The term ‘animal model of preeclampsia’ is commonly and consistently used when nitric oxide synthase inhibitor NG-nitro-L-arginine methylester (L-NAME) was administered from d14 of gestation to induce preeclampsia-like syndrome in rats [40]–[45]. It has been reported that although chronic treatment with L-NAME may not reproduce the entire disease entity, it produces virtually all the pathophysiologies of preeclampsia in the animal model [46]. In view of this the L-NAME induced rat model of pregnancy induced hypertension was used.

### Animals, Breeding and Induction of L-NAME

Wistar albino rats (60 F, 20 M) were used for the present study Out of 60 females, 48 females became pregnant and were randomly divided into control and 5 dietary groups. The six dietary groups (n = 8 per group) were as follows: Control; PIH Induced; PIH Induced + Vitamin B_12_ (excess vitamin B_12_) supplemented group (PIH + B_12_); PIH Induced + Folate supplemented (excess folate) group (PIH + F); PIH Induced + Omega-3fatty acid supplemented group (PIH + O) and PIH Induced + Vitamin B_12_ (excess vitamin B_12_) + Folate (excess folate) + Omega-3 fatty acid supplemented group (PIH + B_12_ + F + O) and have been shown in study design (Table 1).

**Table 1:** Study Design.

Diet groups					
Pre-pregnancy to Pregnancy					
Group 1	Group 2	Group 3	Group 4	Group 5	Group 6
Control	PIH Induced	PIH + Vit B_12_	PIH + F	PIH + O	PIH + F + Vitamin B_12_ + O

Dams were dissected on d20 of gestation (n = 8 from each group); placenta and blood samples were collected.

Dietary Groups: Control; PIH: PIH Induced; PIH + vitamin B_12_; PIH Induced + vitamin B_12_ supplementation; PIH + F: PIH Induced + folate supplementation; PIH + O: PIH Induced + omega 3 fatty acid supplementation; PIH + B_12_ + F + O: PIH Induced + vitamin B_12_ + folate + omega 3 fatty acid supplementation.

All dams were delivered by C section on day 20 of gestation to collect the placenta, liver and brain tissues. Dam blood was collected by cardiac puncture. At the same time pup liver and brain tissues were also collected.

L-NAME was used to induce hypertension in the pregnant rat. The blood pressure of the pregnant dams was recorded on the day L-NAME was administered i.e. d14 of gestation and once again on d19 of gestation. It was observed that L-NAME administration induced maternal hypertension. The dose of LNAME used was 50 mg/kg body weight/day and was administered by gavage from day 14^th^ to 19^th^ of gestation.

### Diet preparation

Diets (control and treatment) (Table 2) were prepared in accordance with the AIN-93 guidelines for purified diets for laboratory rodents [47]. Vitamin-free casein was used for all treatment diets. The composition of diets in each group is given in table 2. The two groups, PIH + O and PIH + B_12_ + F + O were supplemented with omega-3 fatty acids using fish oil capsules (MaxEPA, Merck Darmstadt, Germany) which contained a combination of DHA (120 mg) and eicosapentaenoic acid (EPA) (180 mg) per capsule. The treatment groups PIH + F and PIH + B_12_ + F + O had 8 mg of folic acid per Kg diet; while PIH + B_12_ and PIH + B_12_ + F + O had 50 µg vitamin B_12_ per kg diet (Table 2).

**Table 2:** Diet Composition of Control and Treatment Groups.

Diet components(g/kg)	Control	PIH Induced	PIH + B_12_	PIH + F	PIH + O	PIH + B_12_ + F + O
**Corn Starch**	398	398	398	398	398	398
**Casein**	200	200	200	200	200	200
**Dextrinized Starch**	132	132	132	132	132	132
**Sucrose**	100	100	100	100	100	100
**Soyabean Oil**	70	70	70	70	25	25
**Fish Oil (Maxepa)**	0	0	0	0	45	45
**Fiber**	50	50	50	50	50	50
**Mineral mixture (A)**	35	35	35	35	35	35
**Vitamin mixture (B)**	10	10	10	10	10	10
**Folic acid**	0.002	0.002	0.002	0.008	0.002	0.008
**Vitamin B_12_**	0.025	0.025	0.050	0.025	0.025	0.050
**Cystine**	3	3	3	3	3	3
**Choline chloride**	2.5	2.5	2.5	2.5	2.5	2.5
**Tertiary Butyl**	0.014	0.014	0.014	0.014	0.014	0.014
**Total Energy (kJ)**	15.7	15.7	15.7	15.7	15.7	15.7

A Mineral mixture (g/kg mixture): Calcium carbonate, 357; Potassium Phosphate, 196; Potassium Citrate, 70.78; Sodium Chloride, 78; Potassium Sulphate, 46.6; Magnesium Oxide, 24; Ferric Citrate, 6.06; Zinc Carbonate, 1.65; Manganous Carbonate, 0.63; Cupric Carbonate, 0.3; Potassium Iodate, 0.01; Sodium Selenate, 0.01; Ammonium Paramolybdate, 0.007; Sodium Metasilicate, 1.45; Chromium Potassium Sulphate, 0.275; Lithium Chloride, 0.01; Boric Acid, 0.08; Sodium Fluoride, 0.06; Nickel Carbonate, 0.03; Ammonium Vanadate, 0.006; Sucrose, 221.02.

B Vitamin mixture (g/kg mixture): Nicotinic Acid, 3; Calcium Pantothenate, 1.6; Pyridoxine-HCl, 0.7; Thiamin –HCl, 0.6; Riboflavin, 0.6; D-Biotin, 0.02; Vitamin B_12_ (in 0.1% Mannitol), 2.5; Vitamin E, 15; Vitamin A, 0.8; Vitamin D-3, 0.25; Vitamin K, 0.075; Folic acid, 0.2 (control) and Sucrose 974.655, was used to make total weight of the vitamin mixture to 1 kg.

Dietary Groups: Control; PIH: PIH Induced; PIH + vitamin B_12_; PIH Induced + vitamin B_12_ supplementation; PIH + F: PIH Induced + folate supplementation; PIH + O: PIH Induced + omega 3 fatty acid supplementation; PIH + B_12_ + F + O: PIH Induced + vitamin B_12_ + folate + omega 3 fatty acid supplementation.

### Observations recorded

Feed intake of dams during pregnancy was recorded. During pregnancy, dam weights were recorded at 0, 7, 14 and 20 d to obtain weight gains. On d20 of gestation the litter weight and size was recorded in each group.

### Organ weights

The absolute weights of the brain, liver and placenta were recorded on a Schimadzu electronic balance with a least count of 0.001 g. These vital organs were immediately snap frozen in liquid nitrogen and stored at −80°C for various biochemical estimations. The relative organ weights were expressed as [(organ weight/weight of the animal)*100].

### Analysis of fatty acids

The procedure for fatty acid analysis used in our study was revised from the original method of Manku et al. that has been reported by us earlier in studies [38], [48]. Fatty acids were expressed as g/100 g fatty acid. Total of 15 fatty acids were detected. Saturated fatty acids include myristic acid, palmitic acid and stearic acids, total monounsaturated fatty acids include myristoleic, palmitoleic, oleic and nervonic acids. The omega-3 fatty acids included alpha linolenic acid, eicosapentaenoic acid and docosahexaenoic acid while total omega-6 fatty acids included linoleic acid, gamma linolenic acid, di-homo-gammalinolenic acid, docosapentaenoic acid and arachidonic acid.

### Analysis of Plasma Micronutrients and Homocysteine

Plasma vitamin B_12_, folate and homocysteine levels were determined using commercial kits by the Chemiluminescent Microparticle Immunoassay (CMIA) methods (Abbott Laboratory Abott park, Chicago, IL) on the Abbott Axsym System; 5F51-20 and the method has been reported by us earlier [49], [50]. Plasma vitamin B_12_ levels were expressed as pg/ml, folate levels as ng/ml and homocysteine levels as µmol/L.

### Lipid peroxidation measurements

Oxidative stress marker (MDA) levels were estimated from dam plasma and pup liver using Oxis kits (MDA586, Oxis International, Foster City, CA, USA). Briefly, thiobarbituric acid reacts with MDA to form a pink color, and the absorbance was measured at 586 nm. Tetramethoxypropane is used as a standard. MDA concentration is expressed as nmol/ml.

### Protein carbonyl estimation

Protein carbonyl from pup liver was estimated by the method of Uchida et al. with some modifications [51]. Briefly, 0.5 ml protein samples were mixed with an equal volume of 2, 4-dinitrophenylhydrazine (10 mM) in 2.5 M-HCl and incubated at room temperature for 1 h. After incubation, protein was precipitated by 20% TCA (0·5 ml) and washed three times with 1 ml ethanol: ethyl acetate (1∶1, v/v) mixture. Finally, the precipitate was solubilized in 1 ml of 6 M-urea and absorbance was read at 365 nm. Protein carbonyl concentration was calculated by using the molar extinction coefficient. The results were expressed as nmol carbonyls/mg protein.

### Placental tumor necrosis factor levels-α (TNF- α)

Placental TNF-α were measured using the commercially available specific enzyme linked immunosorbent assay kit (Abcam, Catalog No. ab100785). This assay employs an antibody specific for Rat TNF alpha coated on a 96-well plate. Standards and samples are pipetted into the wells and TNF alpha present in the sample is bound to the wells by the immobilized antibody. The wells are washed and biotinylated anti-rat TNF alpha antibody is added. After washing away unbound biotinylated antibody, HRP-conjugated streptavidin is pipetted into the wells. The wells are again washed, a TMB substrate solution is added to the wells and color develops in proportion to the amount of TNF alpha bound. The stop solution changes the color from blue to yellow, and the intensity of the color is measured at 450 nm. The placental TNF-alpha levels were expressed as pg/ml.

### Blood pressure measurement

The blood pressure of the dams was measured using the pneumatic tail cuff device (IITC Life Science Inc.). The systolic and diastolic BP was recorded on d0, d13 and d19 of gestation for all dams. Three measurements with 30 s intervals were recorded and the average of these readings was calculated.

### Statistical methods

Values were expressed as mean ± SD. In the present study, statistical analyses were performed using one-way analysis of variance (ANOVA), followed by Fisher's LSD test using SPSS/PC+ package (version 20.0 Chicago IL) for windows. A p value less than 0.05 was considered as a statistically significant difference.

## Results

### Feed intake of dams during pregnancy

The feed intake for control and various treatment groups was similar among groups and was as follows: control (14.75±1.45 g/day); PIH induced (15.31±1.60 g/day); PIH + vitamin B_12_ (15.94±1.15 g/day); PIH + F (15.28±0.76 g/day); PE + O (15.60±0.79 g/day) and combined supplementation of folate, vitamin B_12_ and omega-3 fatty acids i.e. PIH + F + Vitamin B_12_ + O (15.19±1.83 g/day).

### Systolic and diastolic blood pressure of dams on d19 of gestation

**Figure 1:**
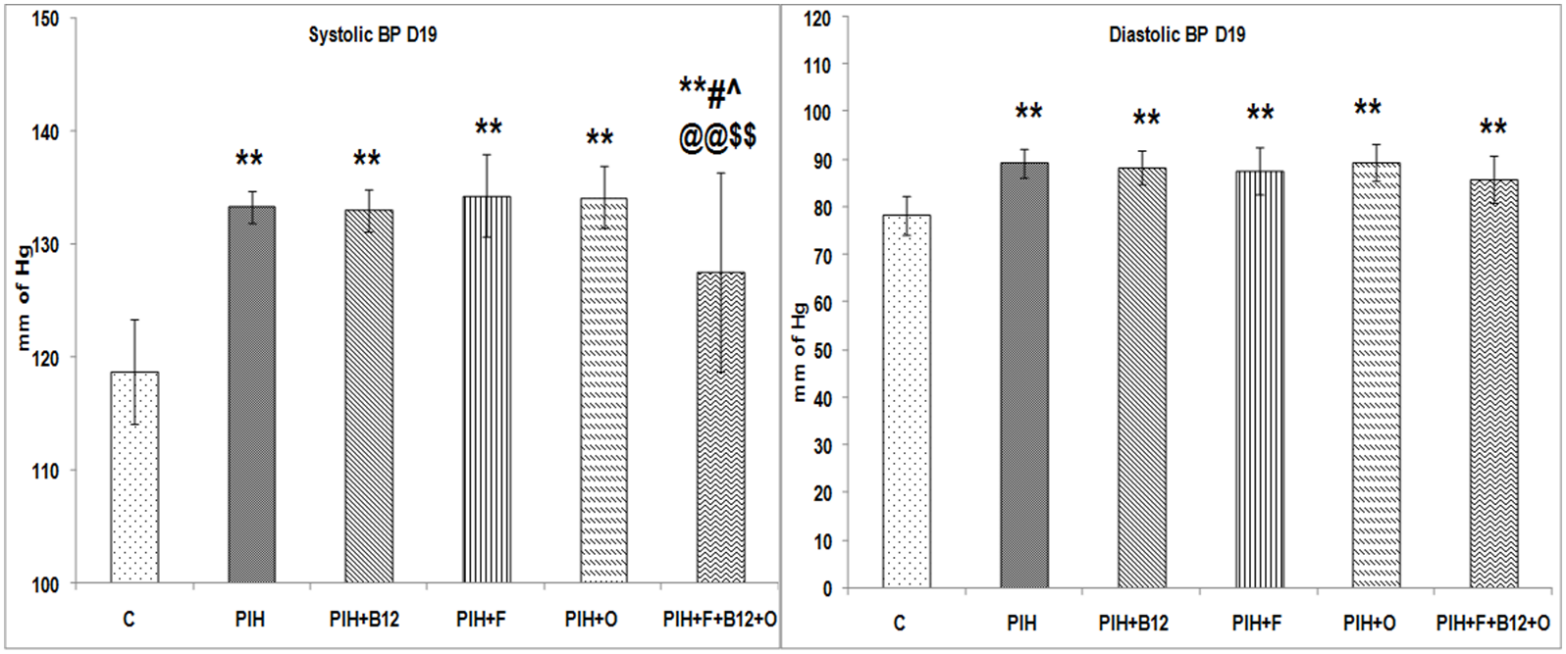
Systolic and Diastolic Blood Pressure Levels in Dams. Values are expressed as Mean ± SD; p: Level of Significance; *p<0.05, **p<0.01 as compared to control; #p<0.05; ##p<0.01 as compared to PIH induced, ∧p<0.05; ∧∧p<0.01 as compared to PIH + B_12_ @p<0.05; @@p<0.01 as compared to PIH + F, $p<0.05; $$p<0.01 as compared to PIH + O.Dietary Groups: Control; PIH Induced; PIH + B_12_: PIH Induced + vitamin B_12_ supplementation; PIH + F: PIH Induced + folate supplementation; PIH + O: PIH Induced + omega 3 fatty acid supplementation; PIH + B_12_ + F + O: PIH Induced + vitamin B_12_ + folate + omega 3 fatty acid supplementation.

The systolic and diastolic BP was similar between groups on d0 of gestation. Both systolic and diastolic BP were higher (p<0.01 for both) on d19 of gestation in the PIH induced group as compared to control. Similarly the systolic and diastolic BP in the maternal vitamin B_12_ (PIH + B_12_), maternal folate (PIH + F) or maternal omega-3 fatty acid supplementation (PIH + O) to PE induced dams was higher (p<0.01 for all) as compared to control. However a combined maternal micronutrient supplementation (PIH + B_12_ + F + O) to PIH induced dams was able to lower only the systolic BP as compared to PIH inducd (p<0.05), PIH + B_12_ (p<0.05), PIH + F (p<0.01) and PIH + O (p<0.01) groups but not as compared to control (Fig. 1).

### Reproductive performance

**Figure 2:**
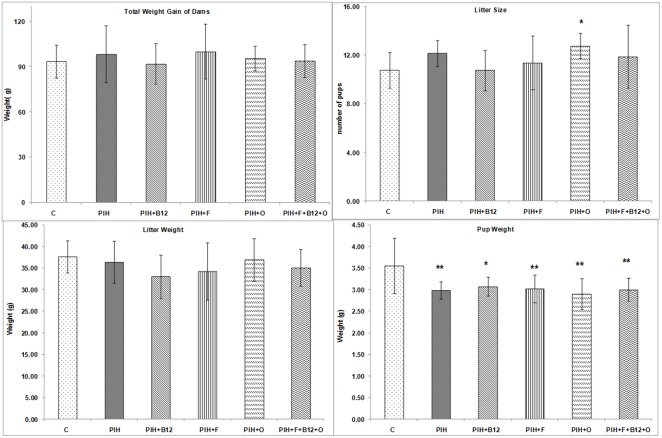
Reproductive Performance, Litter Size and Weight and Pup Weight. Values are expressed as Mean ± SD; p: Level of Significance; *p<0.05, **p<0.01 as compared to control. Dietary Groups: Control; PIH Induced; PIH + B_12_: PIH Induced + vitamin B_12_ supplementation; PIH + F: PIH Induced + folate supplementation; PE + O: PIH Induced + omega 3 fatty acid supplementation; PE + B_12_ + F + O: PIH Induced + vitamin B_12_ + folate + omega 3 fatty acid supplementation.

The total weight gain of dams in the PIH induced group was comparable to control. Similarly supplementation with individual micronutrients like folate (PIH + F), vitamin B_12_ (PIH + B_12_) or omega-3 (PIH + O) fatty acids and a combined micronutrient supplementation (PIH + B_12_ + F + O) also did not affect the weight gains of dams during pregnancy and was comparable to control. The litter size in all the groups was comparable to control. Litter weight is the average weight of all the litters at birth and was not affected by PE induction. In the present study L-NAME administration showed a trend for reduction in litter weight although it did not reach significance. However, the litter size was higher (p<0.05) in the PIH + O group as compared to control. In contrast, PIH induced group showed reduced (p<0.01 for all) pup weight in all groups as compared to control and did not improve either by independent or a combined micronutrient supplementation to PIH induced groups (Fig. 2).

### Organ weights

#### Organ weights of dams on d20 of gestation

In dams, absolute and relative liver weights were comparable between the groups. Omega-3 fatty acid supplementation to PIH induced dams (PIH + O) increased (p<0.05) the absolute as well as relative brain weights as compared to control. Similarly, maternal folic acid supplementation to PIH induced dams (PIH + F) (p<0.05 for all) increased the absolute brain weights as compared to control, PIH induced and PIH + B_12_ groups (Table 3).

**Table 3:** Absolute and Relative Organ Weights of Dams on d19 of Gestation.

	Control(n = 8)	PIH Induced(n = 7)	PIH + B_12_(n = 8)	PIH + F(n = 8)	PIH + O(n = 8)	PIH + B_12_ + F + O(n = 8)
**Absolute Liver Weight (g)**	9.45±1.16	8.89±1.27	8.81±1.08	9.49±0.63	9.06±0.94	9.31±0.58
**Relative liver Weight** **(%)**	3.14±0.36	2.97±0.29	2.96±0.26	3.13±0.14	3.01±0.25	3.14±0.13
**Absolute Brain Weight (g)**	1.76±0.12	1.77±0.09	1.78±0.09	1.90±0.06**^#∧^	1.86±0.07*	1.78±0.12**^@^**
**Relative Brain Weight** **(%)**	0.58±0.04	0.59±0.03	0.60±0.03	0.62±0.02*	0.62±0.02*	0.60±0.02

Values are Mean ± SD; p: Significance; **p<0.01, *p<0.05 as compared to control; ^#^p<0.05 as compared to PIH Induced; ^∧^p<0.05 as compared to PIH + B_12_; ^@^p<0.05 as compared to PIH + F.

Dietary Groups: Control; PIH: PIH Induced; PIH + vitamin B_12_; PIH Induced + vitamin B_12_ supplementation; PIH + F: PIH Induced + folate supplementation; PIH + O: PIH Induced + omega 3 fatty acid supplementation; PIH + B_12_ + F + O: PIH Induced + vitamin B_12_ + folate + omega 3 fatty acid supplementation.

#### Organ weights of offspring at birth

It was observed that pups born to PIH induced dams had lower (p<0.01) absolute liver weights as compared to control. Similarly the absolute liver weight of pups born to dams from PH + F, PIH + O and PIH + B_12_ + F + O groups had lower (p<0.05) liver weights as compared to control. The relative brain weights of pups born to dams from PIH + F, PIH + O, PIH + B_12_ + F + O groups were higher (p<0.05) as compared to control. Absolute and relative placental weights were comparable among all groups (Table 4).

**Table 4:** Absolute and Relative Organ Weights of Offspring at Birth.

	Control(n = 8)	PIH Induced(n = 8)	PIH + B_12_(n = 8)	PIH + F(n = 8)	PIH + O(n = 8)	PIH + B_12_ + F + O(n = 8)
**Absolute Liver Weight (g)**	0.23±0.05	0.16±0.03**	0.19±0.06	0.17±0.03*	0.18±0.04*	0.17±0.05*
**Relative Liver Weight** **(%)**	6.56±1.72	5.51±0.64	6.10±1.70	5.89±1.14	5.93±0.83	5.96±1.23
**Absolute Brain Weight (g)**	0.16±0.02	0.16±0.03	0.16±0.01	0.16±0.02	0.17±0.01	0.16±0.02
**Relative Brain Weight** **(%)**	4.69±0.72	5.35±0.90	5.24±0.92	5.53±0.68*	5.61±0.60*	5.70±0.71**
**Absolute Placenta Weight (g)**	0.41±0.05	0.39±0.09	0.36±0.03	0.36±0.06	0.35±0.04	0.41±0.06
**Relative Placenta Weight (%)**	11.72±2.22	13.12±2.86	11.57±1.65	12.00±2.19	11.92±2.54	13.85±2.02

Values are Mean ± SD; p: Significance; **p<0.01, *p<0.05 as compared to control.

Dietary Groups: Control; PIH: PIH Induced; PIH + vitamin B_12_; PIH Induced + vitamin B_12_ supplementation; PIH + F: PIH Induced + folate supplementation; PIH + O: PIH Induced + omega 3 fatty acid supplementation; PIH + B_12_ + F + O: PIH Induced + vitamin B_12_ + folate + omega 3 fatty acid supplementation.

### Dam plasma vitamin B_12_, folate and homocysteine levels on d20 of gestation

The levels of plasma vitamin B_12_ were comparable to control in the PIH induced group while that of folate was lower (p<0.01) as compared to control. In contrast the levels of plasma homocysteine were higher (p<0.05) in the PIH induced group as compared to control. Maternal vitamin B_12_ supplementation to PIH induced (PIH + B_12_) group increased (p<0.01) the levels of plasma vitamin B_12_. However, it could not normalize the levels of homocysteine and lowered (p<0.05) the plasma folate levels as compared to control.

Maternal folic acid supplementation to PIH induced (PIH + F) dams normalized the levels of folic acid to that of control. However, it also could not normalize the plasma homocysteine levels as compared to control although vitamin B_12_ levels were similar to that of control. Maternal omega-3 fatty acid supplementation to PIH induced dams (PIH + O) increased (p<0.01 for both) the levels of plasma vitamin B_12_ but did not normalize the homocysteine although levels of plasma folate were comparable to control.

**Figure 3:**
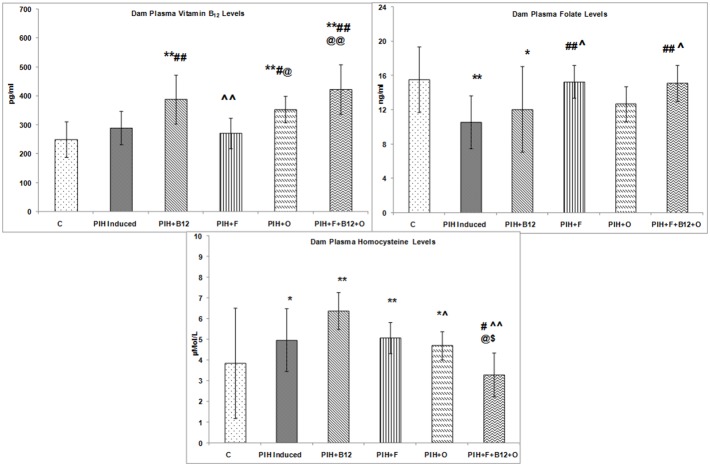
Dam Plasma Vitamin B_12_, Folate and Homocysteine Levels. Values are expressed as Mean ± SD; p: Level of Significance; *p<0.05, **p<0.01 as compared to control; #p<0.05; ##p<0.01 as compared to PIH induced, ∧p<0.05; ∧∧p<0.01 as compared to PIH + B_12_ @p<0.05; @@p<0.01 as compared to PIH + F, $p<0.05; $$p<0.01 as compared to PIH + O. Dietary Groups: Control; PIH Induced; PIH + B_12_: PIH Induced + vitamin B_12_ supplementation; PIH + F: PIH Induced + folate supplementation; PIH + O: PIH Induced + omega 3 fatty acid supplementation; PIH + B_12_ + F + O: PIH Induced + vitamin B_12_ + folate + omega 3 fatty acid supplementation.

A combined maternal micronutrient supplementation to PIH induced dams (IH + B_12_ + F + O) increased (p<0.01) the levels of plasma vitamin B_12_ and normalised the levels of homocysteine to that of control. Levels of plasma folate in this group were also comparable to control (Fig. 3).

### Placental fatty acid levels on d20 of gestation

**Figure 4:**
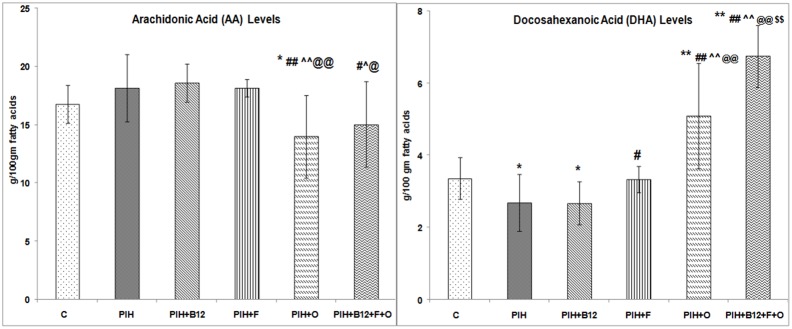
Levels of Arachidonic Acid and Docosahexaenoic Acid Levels in the Dam Placenta on d20 of Gestation. Values are expressed as Mean ± SD; p: Level of Significance; *p<0.05, **p<0.01 as compared to control; #p<0.05, ##p<0.01 as compared to PIH induced, ∧p<0.05; ∧∧p<0.01 as compared to PIH + B_12_; @p<0.05; @@p<0.01 as compared to PIH + F, $$p<0.01 as compared to PIH + O. Dietary Groups: Control; PIH Induced; PIH + B_12_: PIH Induced + vitamin B_12_ supplementation; PIH + F: PIH Induced + folate supplementation; PIH + O: PIH Induced + omega 3 fatty acid supplementation; PIH + B_12_ + F + O: PIH Induced + vitamin B_12_ + folate + omega 3 fatty acid supplementation.

The placental DHA levels were lower (p<0.05) while levels of arachidonic acid (AA) were similar to control in the PIH induced group. Maternal vitamin B_12_ supplementation to PIH induced dams (PIH + B_12_) also showed lower (p<0.05) DHA levels as compared to control. Maternal folate supplementation to PIH induced dams (PIH + F) was able to normalise levels of DHA as compared to control but showed higher (p<0.05 for both) DHA levels as compared to PIH induced group. Maternal omega-3 fatty acid supplementation to PIH induced dams (PIH + O) lowered (p<0.01 for all) the levels of AA and increased (p<0.01 for all) DHA as compared to control, PIH, PIH + B_12_ and PIH + F groups in the placenta. A combined maternal micronutrient supplementation (PIH + B_12_ + F + O) also lowered (p<0.05 for all) the levels of AA as compared to PIH, PIH + B_12_ and PIH + F groups. In contrast levels of DHA in the placenta in this group were higher (p<0.01 for all) as compared to all other treatment groups (Fig. 4).

### Dam plasma MDA levels on d20 of gestation

**Figure 5:**
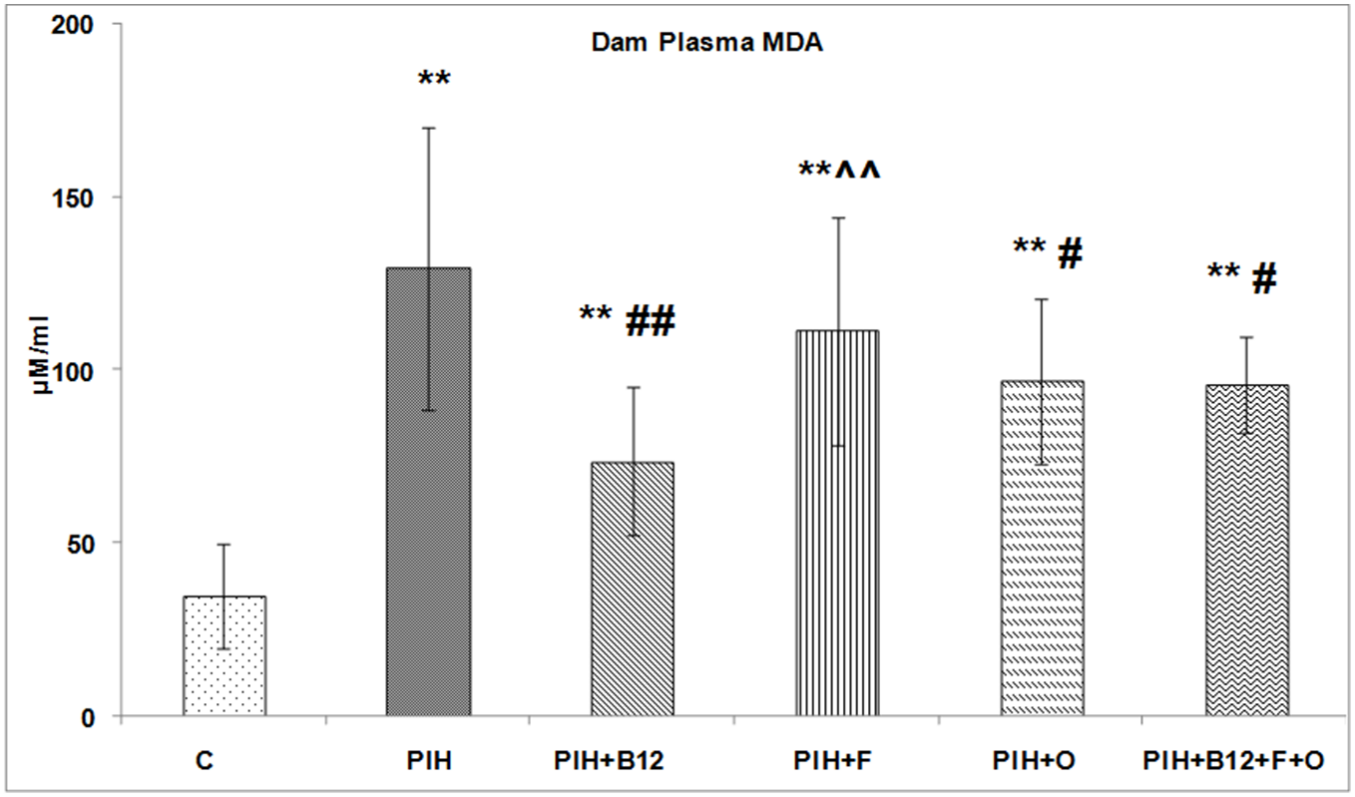
Dam Plasma MDA Levels. Values are expressed as Mean ± SD; p: Level of Significance; **p<0.01 as compared to control; #p<0.05; ##p<0.01 as compared to PIH induced; ∧∧p<0.01 as compared to PIH + B_12_. Dietary Groups: Control; PIH Induced; PE + B_12_: PIH Induced + vitamin B_12_ supplementation; PIH + F: PIH Induced + folate supplementation; PIH + O: PIH Induced + omega 3 fatty acid supplementation; PIH + B_12_ + F + O: PIH Induced + vitamin B_12_ + folate + omega 3 fatty acid supplementation.

PE induction increased (p<0.01) the plasma MDA levels as compared to control. In contrast, maternal vitamin B_12_ supplementation (PIH + B_12_), maternal omega-3 fatty acid supplementation (PIH + O) or a combined maternal micronutrient supplementation (PIH + B_12_ + F + O) to PIH induced dams was able to lower (p<0.05 for all) the plasma MDA levels as compared to PIH group but not as compared to control (Fig. 5).

### Placental tumor necrosis factor –α (TNF-alpha) levels on d20 of gestation

**Figure 6:**
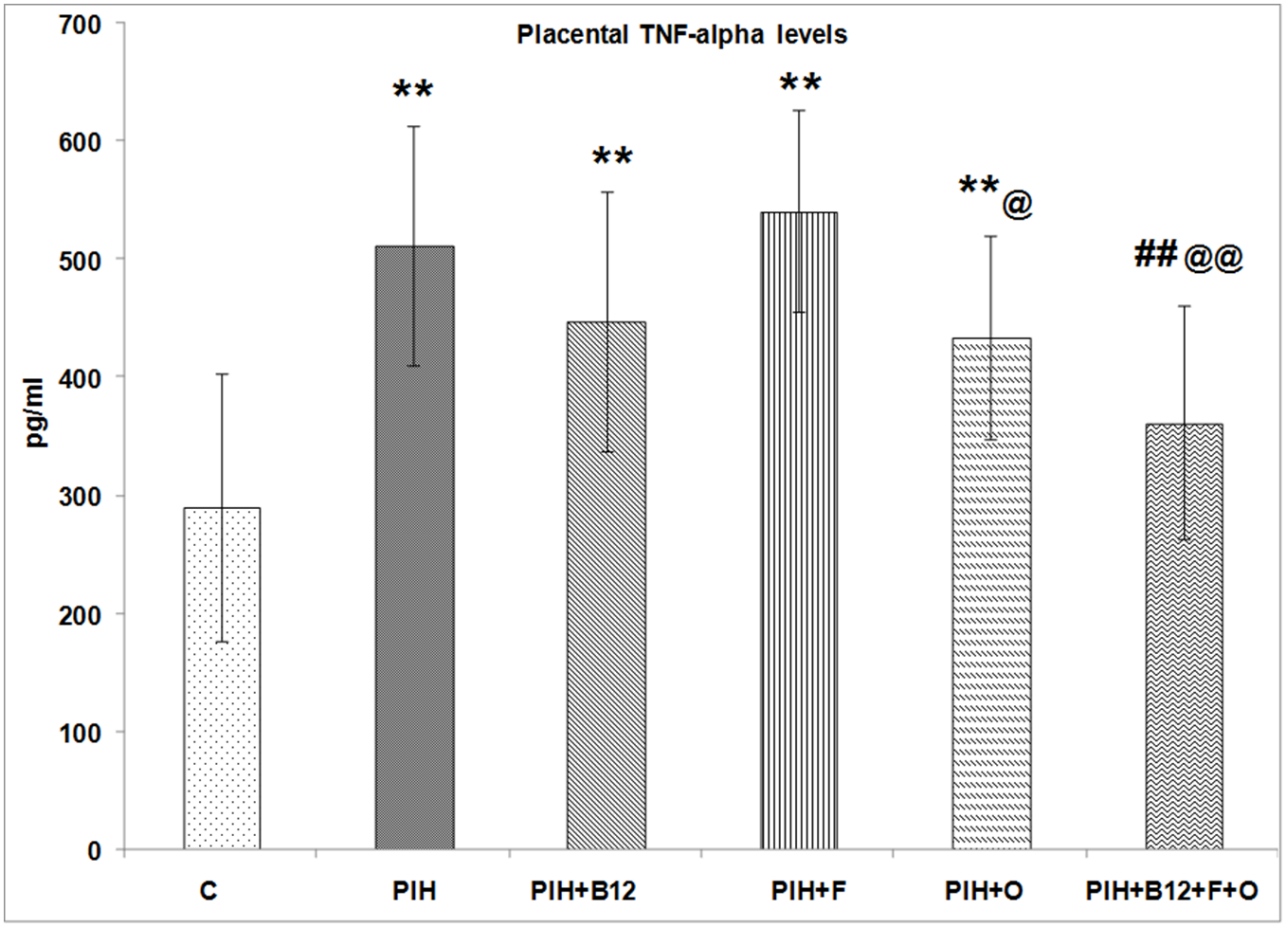
Dam Placental TNF alpha Levels. Values are expressed as Mean ± SD; p: Level of Significance; **p<0.01 as compared to control; ##p<0.01 as compared to PIH induced; @p<0.05; @@p<0.01 as compared to PIH + F. Dietary Groups: Control; PIH Induced; PIH + B_12_: PIH Induced + vitamin B_12_ supplementation; PIH + F: PIH Induced + folate supplementation; PIH + O: PIH Induced + omega 3 fatty acid supplementation; PIH + B_12_ + F + O: PIH Induced + vitamin B_12_ + folate + omega 3 fatty acid supplementation.

PIH induced dams showed higher (p<0.01) levels of placental TNF- α as compared to control. Supplementation with individual micronutrients did not offer any benefit since maternal vitamin B_12_ (PE + B_12_), maternal folate (PE + F), maternal omega-3 fatty acid supplementation to PIH induced dams also showed higher (p<0.01 for all) TNF- α levels in the placenta as compared to control. In contrast, a combined maternal micronutrient supplementation (PIH + B_12_ + F + O) to PIH induced dams was able to lower (p<0.01 for both) the levels of TNF- α levels in the placenta as compared to PIH and was comparable to that of control (Fig. 6).

### Liver oxidative stress indices in the offspring at birth

**Figure 7:**
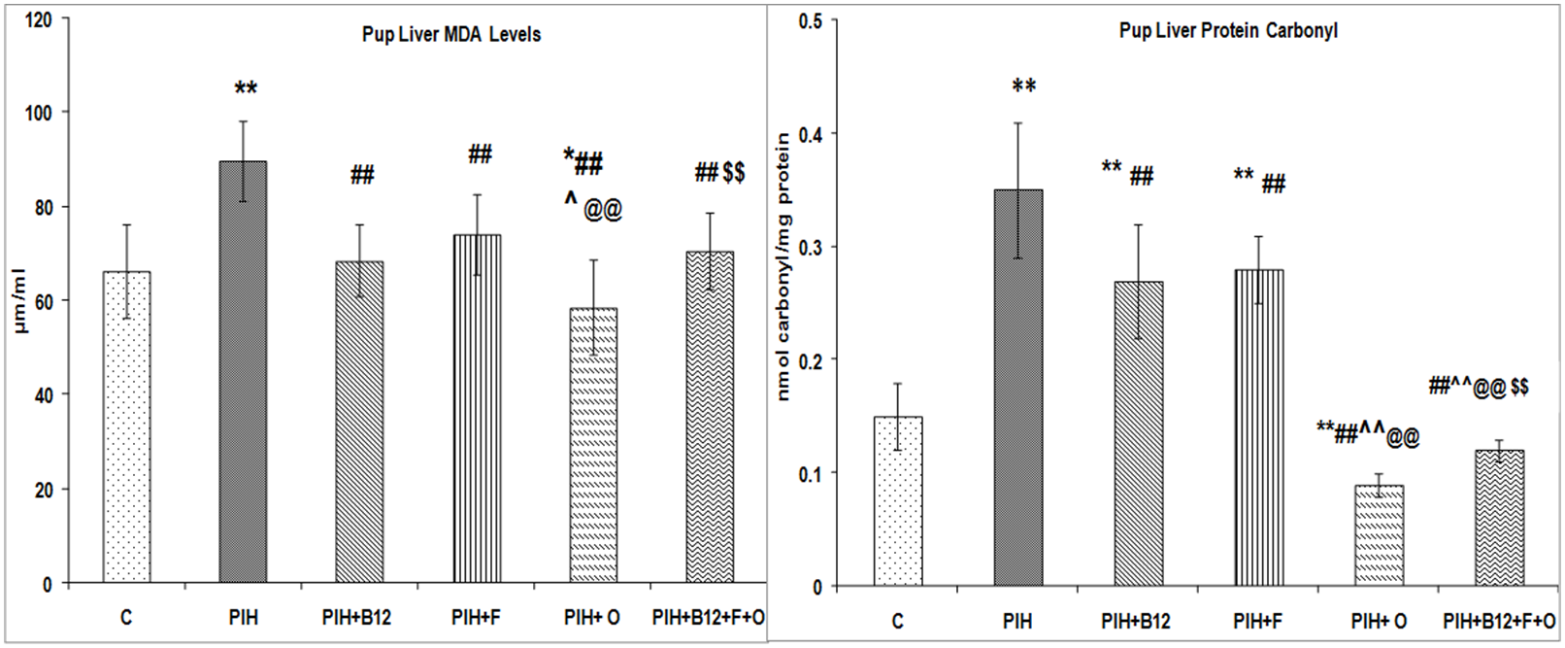
Liver Oxidative Stress Indices in the Offspring at Birth. Values are Mean ± SD; **p<0.01 as compared to C, ##p<0.01 as compared to PIH induced, @@p<0.01as compared to PIH + F; $$p<0.01 compared to PIH + O; ∧p<0.05, ∧∧p<0.01 as compared to PIH + B_12_. Dietary Groups: Control; PIH Induced; PIH + B_12_: PIH Induced + vitamin B_12_ supplementation; PIH + F: PIH Induced + folate supplementation; PIH + O: PIH Induced + omega 3 fatty acid supplementation; PIH + B_12_ + F + O: PIH Induced + vitamin B_12_ + folate + omega 3 fatty acid supplementation.

The pup liver MDA and protein carbonyl levels were higher (p<0.01 for both) in the PIH group as compared to control. Maternal vitamin B_12_ supplementation (PIH + B_12_), and maternal folate (PIH + F) to PIH induced dams lowered (p<0.01 for both) the liver MDA levels in offspring as compared to the PIH group and were comparable to control. Similarly the protein carbonyl levels in these groups were also lower (p<0.01 for both) as compared to PIH but remained higher (p<0.01 for both) as compared to control. In contrast a maternal omega-3 fatty acid (PE + O) supplementation to PIH induced dams was able to lower (p<0.01 for all) the liver MDA and protein carbonyl levels as compared to control and other groups. Similarly a combined maternal micronutrient supplementation (PIH + B_12_ + F + O) to PIH induced dams also normalized the levels of liver MDA and protein carbonyl in offspring to that of control and PIH groups (Fig. 7).

### Liver fatty acid levels in the offspring at birth

**Figure 8:**
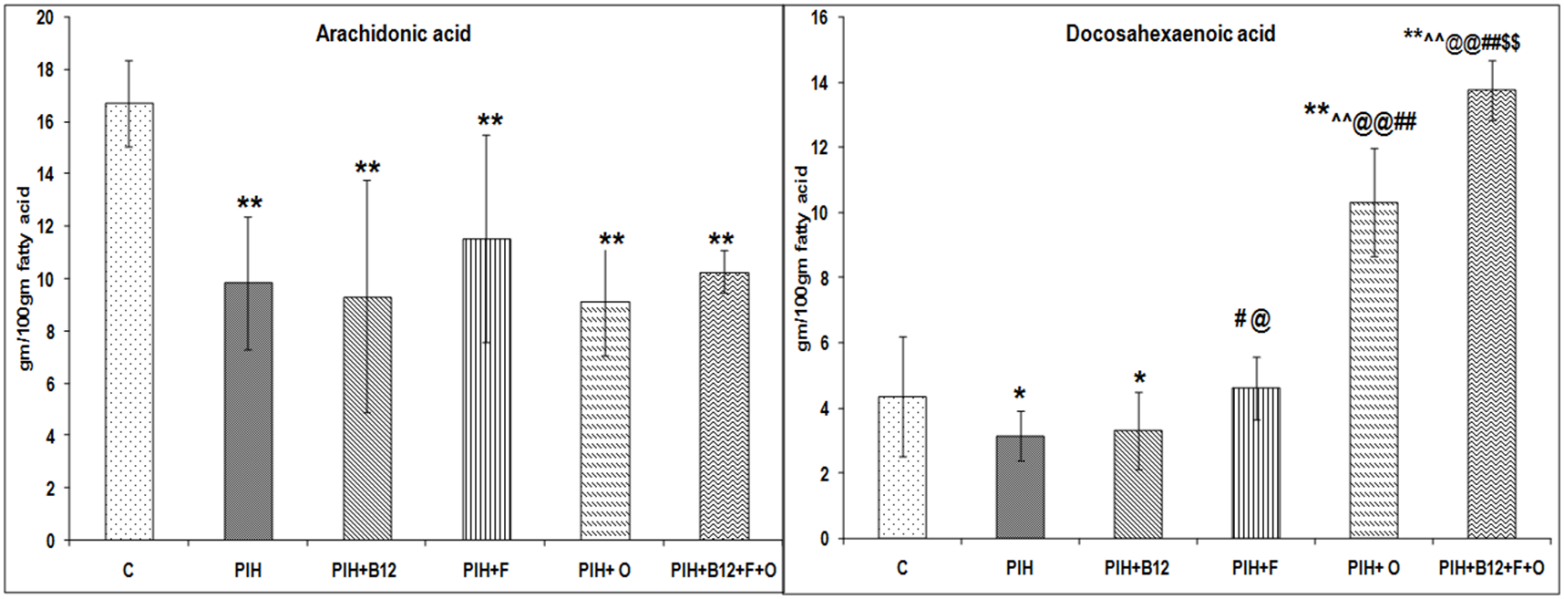
Levels of Arachidonic Acid and Docosahexaenoic in the Offspring Liver at Birth. Values are Mean ± SD; **p<0.01 as compared to C, # p<0.05, ##p<0.01, as compared to PIH induced, @@p<0.01 as compared to PIH + F; $$p<0.01 compared to PIH + O; ∧∧p<0.01 as compared to PIH + B_12_. Dietary Groups: Control; PIH Induced; PIH + B_12_: PIH Induced + vitamin B_12_ supplementation; PIH + F: PIH Induced + folate supplementation; PIH + O: PIH Induced + omega 3 fatty acid supplementation; PIH + B_12_ + F + O: PIH Induced + vitamin B_12_ + folate + omega 3 fatty acid supplementation.

The levels of AA (p<0.01) and DHA (p<0.05) in the liver of the offspring born to PIH induced dams were lower as compared to control. Maternal vitamin B_12_ supplementation to PIH induced (PIH + B_12_) dams showed lower liver AA (p<0.01) and DHA (p<0.05) in the offspring as compared to control. In contrast maternal folate supplementation to PIH induced dams was able to improve (p<0.05) DHA levels in the liver of the offspring as compared to control and PIH group while levels of AA continued to remain low (p<0.01) in this group as compared to control. Similarly maternal omega-3 fatty acid supplementation (PIH + O) as well as combined maternal micronutrient supplementation (PIH + B_12_ + F + O) was able to normalize the levels of DHA to that of control (Fig. 8).

## Discussion

This study for the first time demonstrates the effects of either an individual or a combined maternal micronutrient (folic acid and vitamin B_12_) and omega-3 fatty acid supplementation on placental fatty acids, inflammatory cytokines and blood pressure in a rat model of pregnancy induced hypertension on d20 of gestation. The key findings indicate that PIH induction 1) increases systolic as well as diastolic blood pressure 2) lowers pup weight 3) increases the dam plasma and pup liver oxidative stress 4) increases placental TNF alpha levels and 5) lowers placental and pup liver DHA levels. These effects of PE induction were ameliorated by a combined supplementation of folate, vitamin B_12_ and omega-3 fatty acids. In the present study, PIH induction using L-NAME administration increased blood pressure and is consistent with other recent reports [52]–[55]. However, individual supplementation of folic acid, vitamin B_12_ or omega-3 fatty acids did not lower while a combined supplementation was able to normalize the systolic blood pressure. Animal and human studies indicate that long chain polyunsaturated fatty acid supplementation reduces blood pressure [56], [57]. Similarly, in humans it has been reported that supplementation of multivitamins containing folic acid is associated with reduced risk of preeclampsia [17], [18]. Also, higher folate intake in young adulthood is reported to be associated with a lower incidence of hypertension later in life [58]. On the other hand reports indicate that vitamin B_12_ supplementation in older people with elevated baseline homocysteine concentrations did not lower BP [59]. Earlier it was shown that n-3 fatty acids provided in the third trimester of normal pregnancy did not influence blood pressure [60].

In the present study PIH induction did not affect the total weight gain of dams during pregnancy in different groups and was comparable to control. This is similar to earlier reported studies [61], [62]. In contrast others report lower body weights of dams on d18 of gestation as compared to control [63].

The present study also showed no effect of PIH induction on litter size and litter weight. In contrast, a few studies have shown reduced litter size in PE induced animals [45], [63]–[64]. Further in the present study L-NAME administration showed lower pup weights suggesting that intrauterine growth retardation may a consequence of severe preeclampsia. Our findings are in line with other studies reporting lower fetal weights in L-NAME treated dams [65]–[67]. In contrast other studies report higher fetal weights [68] or no change in fetal weights [69].

Studies using rat model of pregnancy induced hypertension and examining the effect of micronutrient supplementation are limited. In our study maternal supplementation of either folate, vitamin B_12_, DHA or a combination of folate, vitamin B_12_ and DHA did not affect the litter size, litter weight and pup weights.

In the present study L-NAME administration increased homocysteine levels and is similar to our human studies which report increased homocysteine levels in women with preeclampsia [38]. Reports indicate that elevated levels of serum homocysteine may be associated with severity of pre-eclampsia [12], [70]–[72]. In the present study only a combined supplementation of vitamin B_12_, folate and omega-3 fatty acids was able to normalize the homocysteine levels. Earlier, studies indicate that folate, vitamin B_12_ and B_6_ supplementation for 6 weeks in women with pregnancy complication reduces homocysteine levels [73]. Higher homocysteine levels are associated with increased oxidative stress [74]. It has been reoprted that hyperhomocysteinemia is associated with oxidative stress [75] and is proposed to play a role in the pathogenesis of preeclampsia [70], [76]. In the present study elevated MDA levels were observed in the PE induced group. Oxidative stress may play a central role in the pathophysiology of preeclampsia and higher levels have been reported by us and others in human [38], [39], [77]–[80] and animal studies [67], [68]. It has been reported that increased free radicals lead to cellular dysfunction, oxidative damage of biomolecules and endothelial dysfunction [81]. A recent review highlights the need to supplement preeclamptic women with antioxidants with a combination of essential fatty acids (eicosapentaenoic acid and docosahexaenoic acid) during pregnancy to counteract oxidative stress to prevent or delay the onset of preeclampsia and improve the health of mother and baby [27].

In present study, combined micronutrient supplementation was able to reduce the oxidative stress by lowering plasma MDA levels. It has been suggested that an antioxidant- micronutrient cocktail can modulate biomarkers of oxidative stress and inflammation in humans [82]. In addition a recent animal study demonstrates that ω-3 PUFA supplementation reduces placental oxidative stress and enhances placental and fetal growth [83].

Preeclampsia is considered to have a multifactorial etiology associated with inflammatory dysfunction [84]. In the present study, higher levels of placental TNF-alpha was observed in PIH induced dams and is similar to earlier reported human studies [85]–[89] as well as in animals [90]. In contrast there are some studies which show no significant differences in the serum levels of TNF-alpha between control and preeclamptic patients [91]–[93].

In the present study L-NAME administration from d14 of gestation increased the levels of placental TNF alpha levels. These levels were not normalized when the L-NAME induced dams were supplemented with individual micronutrients. Thus only a combined micronutrient supplementation to L-NAME induced dams would be able to normalise the levels of placental TNF alpha. Reports indicate that fish oil has antioxidant, anti-inflammatory and anti-apoptotic properties [94]. It has been reported that n-3 PUFAs imparts their anti-inflammatory effects via reduction of the transcription factor nuclear factor-κB activation which is a potent inducer of proinflammatory cytokine like tumor necrosis factor-α [95]. Alternatively n-3 PUFAs are suggested to repress lipogenesis and increase resolvins and protectin generation, ultimately leading to reduced inflammation [83]. Omega 3 fatty acids are reported to reduce the production of proinflammatory cytokines [96].

In the present study PIH induction lowered placental DHA levels. These findings are in line with our earlier studies in women with PE [97] and also preterm pregnancy [48] which have reported lower DHA levels in the placenta. In present study a combined micronutrient supplementation increased DHA levels but also lowered arachidonic acid (AA) levels. Inverse relation between omega-3 fatty acids and risk of preeclampsia has been reported earlier [25], [98].

Many human and animal studies have linked oxidative stress and prenatal hypoxia to the fetal programming of adult diseases in the offspring [99]–[102] through the epigenetic processes [103]. In present study, increased MDA levels were observed in offspring born to PIH induced dams which is consistent with other human studies [104], [105]. We have earlier reported higher oxidative stress and lower antioxidant levels in cord samples of preeclamptic women [39]. Also in present study, protein carbonyl levels were higher in these offspring which is in agreement with other human reported studies [106], [107].

In present study a combined micronutrient supplementation was beneficial in lowering the levels of MDA and protein carbonyl in offspring born to rat model of pregnancy induced hypertension. One limitation of the study was that proteinuria was not measured. Nevertheless other studies have also reported maternal hypertension using administration of L-NAME with no reports on proteinuria [45], [61].

**Figure 9:**
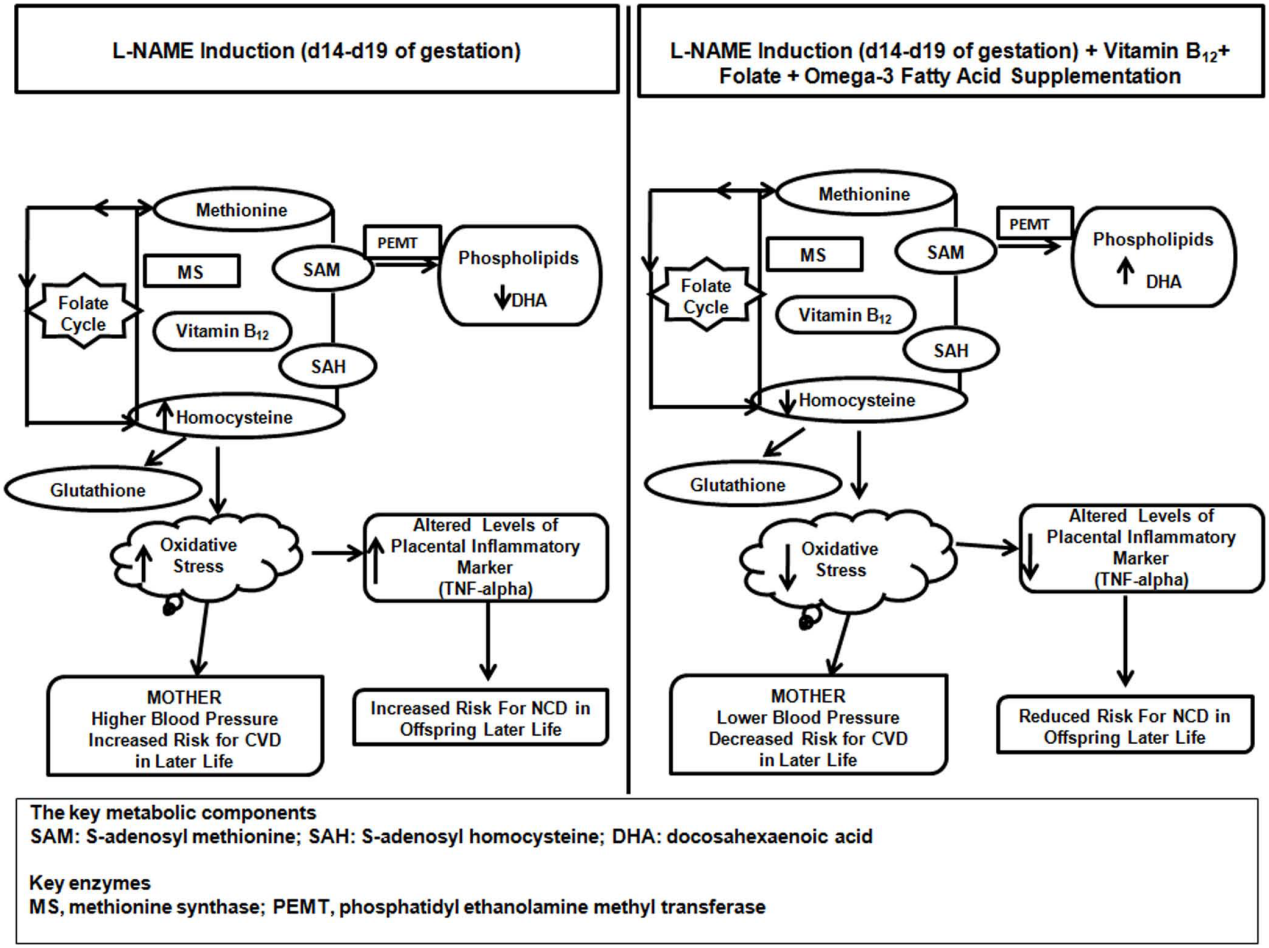
Schematic Representation of Effect of Combined Micronutrient Supplementation to L-NAME induced Dams on Risk for Non Communicable Diseaeses in Offspring in Later Life.

In the present study it is clearly seen that L-NAME administration to pregnant dams increases oxidative stress in both dams and offspring at birth. A combined supplementation of folate, vitamin B_12_ and omega-3 fatty acids was able to reduce the oxidative stress in both dams and offspring as compared to the L-NAME treated group. We have elaborately discussed that micronutrients (folic acid and Vitamin B_12_) and DHA are interlinked in the one carbon cycle in a series of human and animal studies [38], [49], [50], [108], [109]. Changes in any of these nutrients can affect homocysteine levels, oxidative stress and also methylation reactions. Our human studies in women with preeclampsia have also demonstrated a negative association of DHA (an omega 3 fatty acid) with homocysteine levels [38]. In our recent article we have elaborately described the possibility of ameliorating oxidative stress during pregnancy by modulation of the maternal one carbon cycle [110]. Thus, in the present study, synergistic effects of these combined nutrients have beneficial effects in reducing the severity of preeclampsia while individual micronutrient supplementation did not provide much benefit in terms of reducing severity (Fig. 9).

## Conclusion

To summarize, PIH induced dams demonstrated higher systolic and diastolic blood pressure, lower pup weight; increased oxidative stress makers (plasma homocysteine and malondialdehyde (MDA) levels), lower placental docosahexaenoic acid (DHA) and increased inflammatory marker, tumor necrosis factor –alpha (TNF –alpha) levels as compared to control. These findings are in accordance with our human study where in PE women maternal oxidative stress homocysteine and DHA levels were shown to affect angiogenesis and contribute to the preeclamptic pathology and result in adverse effects on fetal growth measures. It has been reported that some of the clinical manifestations in preeclampsia may be a result of alterations in inflammatory mechanisms. Our study suggests that increased oxidative stress may contribute to placental inflammation which may lead to endothelial dysfunction resulting in preeclampsia.

In the current study, individual micronutrient supplementation did not offer much benefit. We have earlier reported interaction of micronutrients (folic acid, vitamin B_12_) and DHA in the one carbon cycle and altered global DNA methylation patterns in human preeclamptic placenta. In the present study, a combined micronutrient supplementation to PIH induced showed beneficial effects in terms of reducing blood pressure, inflammation and oxidative stress. We also demonstrated that pup liver MDA and protein carbonyl levels were higher in the offspring born to PIH induced dams and a combined micronutrient supplementation showed beneficial effects.

To conclude the findings of the current study may have relevance to PE suggesting that combined supplementation of folic acid, vitamin B_12_ and omega-3 fatty acids may have implications for reducing oxidative stress and inflammation in preeclampsia. This may help to ameliorate the risk for non communicable diseases in the offspring.
